# A case report of REM sleep behavior disorder, Behcet’s disease, Sjogren’s syndrome and cognitive dysfunction

**DOI:** 10.1186/s41927-018-0022-y

**Published:** 2018-07-02

**Authors:** Fulong Xiao, Jun Zhang, Waner Wang, Xuehua Wang, Wei Zhang, Liyue Xu, Fang Han

**Affiliations:** 1 Sleep Medicine Center, Department of Respiratory and Critical Care Medicine, Peking University People’s Hospital, Beijing, 100044 People’s Republic of China; 2 Department of Neurology, Peking University People’s Hospital, Beijing, 100044 People’s Republic of China; 3 PKU-Upenn Sleep Center, Peking University International Hospital, Beijing, 102206 People’s Republic of China

**Keywords:** REM sleep behavior disorder, Behcet’s disease, Sjogren’s syndrome

## Abstract

**Background:**

Behcet’s disease and Sjogren’s syndrome is an autoimmune disorder from which many systems of the body can suffer. Here we reported a patient with a history of Behcet’s disease and Sjogren’s syndrome in which REM sleep behavior disorder (RBD) was then detected by polysomnographic (PSG) monitoring.

**Case presentation:**

A 68-year-old male patient with a history of Behcet’s disease and Sjogren’s syndrome was diagnosed with RBD by clinical examination and video-PSG, and he also underwent a multiple sleep latency test and cerebral magnetic resonance imaging. The patient had a history of Behcet’s disease for 20 years and Sjogren’s syndrome for 2 years. The cerebral magnetic resonance imaging also suggested cerebral demyelination and mild cortical atrophy, with cognitive dysfunction by a score of 28 on the mini-mental state examination (MMSE) and a score of 22 on the Montreal cognitive assessment (MoCA).

**Conclusion:**

RBD is common in the elderly population and is significantly related to α-synucleinopathy. Combining the decline in neuro-cognition and mild cortical atrophy, presentation of RBD in this patient could indicate an underlying α-synucleinopathy neurodegenerative disorder in the future. Considering the role of inflammation in the pathogenesis of α-synucleinopathy and a common shared HLA allelic genes in RBD and Sjogren’s syndrome, it is suggested that a physiological process which is related to neuroinflammation may be involved in the pathogenesis of RBD.

## Background

Behcet’s disease is especially common in Middle Eastern and Mediterranean countries. It is an autoimmune disorder from which many systems of the body can suffer [1]. The disease is characterized by oral and genital aphthous ulcers and ocular problems, including uveitis, retinal vasculitis and skin lesions. The Mason-Barnes diagnostic criteria for Behcet’s disease contain at least three of the major signs (1. oral and genital ulcers; 2. ocular lesions, mainly uveitis; 3. cutaneous lesions) or the combination of two major signs above and one minor sign (gastrointestinal or central nervous system and/or peripheral nervous system involvement, arterial lesions, thrombophlebitis, arthritis and familiar history) [2]. Sjogren’s syndrome is also an autoimmune disorder. Xerophthalmia and xerostomia are the two main clinical manifestations of Sjogren’s syndrome [2]. Salivary gland biopsy shows extensive lymphocytic infiltration. The spectrum of central nervous system (CNS) manifestations in Sjogren’s syndrome includes meninges, brain parenchyma and brain vessels [2]. Both the Behcet’s disease and Sjogren’s syndrome can induce vasculitis in the CNS, and complication of Sjogren’s syndrome may exacerbate the exiting collagen vascular pathology [2].

REM sleep behavior disorder (RBD), first described in 1986 [3], is a parasomnia characterized by repeated episodes of dream enactment behavior and REM sleep without atonia (RWA), detected during polysomnographic (PSG) recording and manifested as increased phasic and/or tonic muscle activity on electromyogram channels. RBD may be idiopathic or symptomatic (secondary), strongly associated with neurodegenerative disease [4–7]. It is considered idiopathic RBD in the absence of any other known disorders, although some investigators categorize RBD resulted from medication (particularly antidepressants) as secondary RBD [8]. Literature reports showed that a high proportion, 47.9–75% of patients were secondary RBD [9–11]. The neurodegenerative diseases associated with RBD are synucleinopathies such as Parkinson’s disease, Lewy body disease or dementia with Lewy bodies and multiple systems atrophy [12], although the exact mechanisms are yet unknown.

Here we report a male patient who suffered from two decades of Behcet’s disease in which RBD was diagnosed by PSG combined with cerebral infarction.

## Case presentation

A 68 years old male patient was referred to our sleep center because of 6-year history of falling from bed during sleep and 3-year history of repeated nocturnal episodes of violent and complex behaviors clearly reflecting dream enactment with frequent dream recall (e.g. being chase by dog, jumping over a wall, etc). During the episodes, the patient often screamed, fell from the bed, and would injure his wife. The episodes were reported to recur 2–3 times per month at the time of our evaluation. In addition, the patient reported infrequent episodes of discomfort in the lower limbs during sleep, which often cause awaking in the nocturnal sleep, together with excessive daytime somnolence. Finally, the patient also reported a deficiency in recent memory (the duration was not remembered and it was noticed by the patient’s wife and daughter).

The male patient had a history of Behcet’s disease for 20 years and a history of Sjogren’s syndrome for 2 years before the sleep disorder was reported, and the immune diseases were treated by Total glycosides of paeony (TGP), loxoprofen and mycophenolate mofetil.

**Fig. 1:**
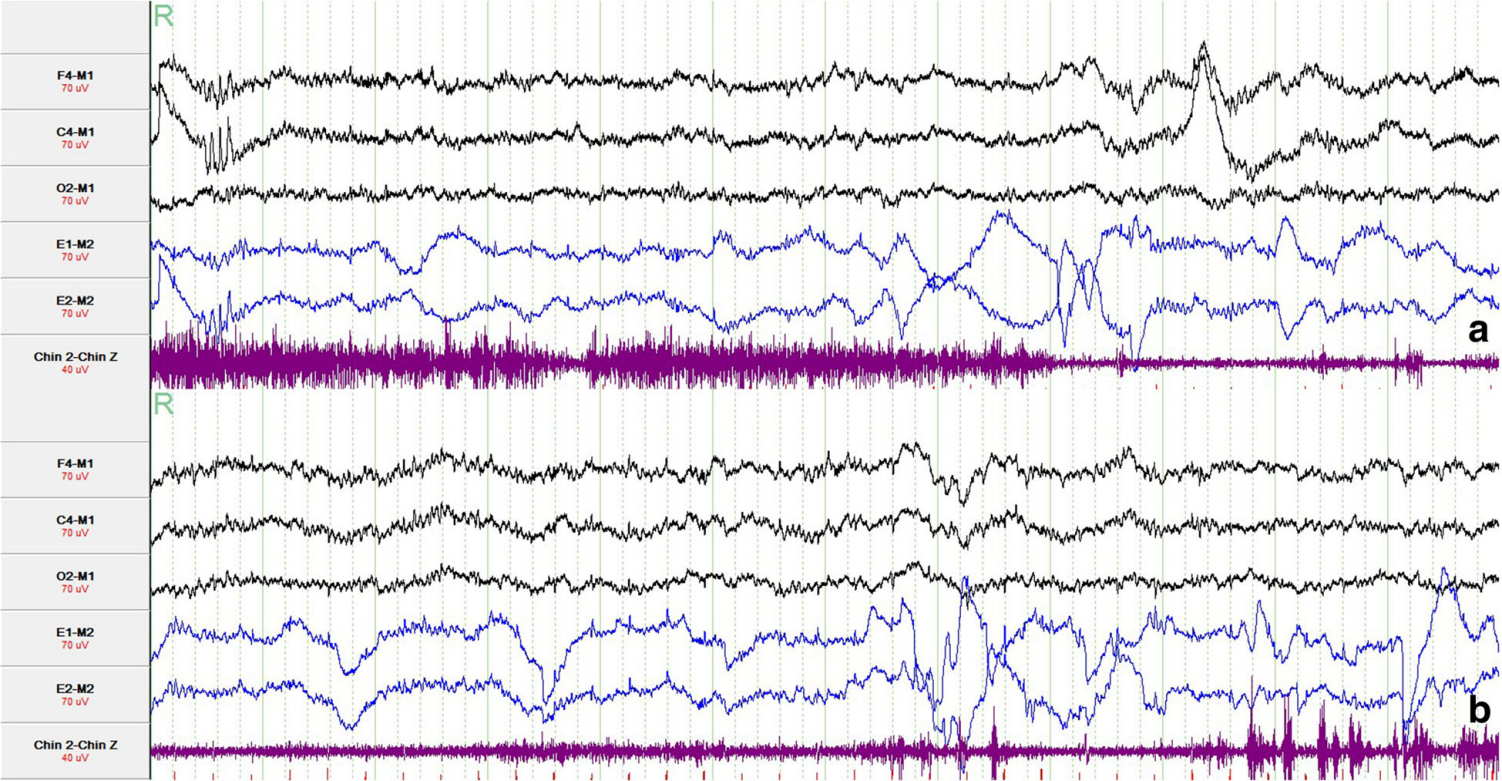
PSG showed tonic chin electromyogram (1a and 1b) activations during REM sleep

Neurological examination showed a weakness in the left limbs (muscle strength IV grade) and deep tendon hyperreflexia in the left lower limb without extrapyramidal signs. Neuropsychological examination showed a score of 28/30 on the mini-mental state examination (MMSE) and a score of 22/30 on the Montreal cognitive assessment (MoCA), with a score of 18 (> 10) on the Epworth sleepiness scale (ESS). Nocturnal video PSG disclosed a sleep latency of 6 min, REM latency of 139 min, sleep efficiency of 92.8%, and abnormal representation of the different sleep stages, with increased N1% sleep period time (SPT) 35.9%, decreased N2% SPT 49.1%, decreased N3% SPT 0.1% and decreased REM% SPT 14.8%; periodic leg movement during sleep (PLMS) index was 64.5/h; periodic leg movements were less in REM sleep (total 20 times) than in NREM sleep (total 502 times) and did not show a significant correlation with arousals. The arousal index was 18/h and times of arousals were less in REM sleep (total 19 times) than in NREM sleep (total 127 times), with the respiratory associated arousal index 5.7/h, PLM associated arousal index 1.1 and spontaneous arousal index 11.2/h. The sleep respiratory pattern was mild abnormal (apnea/hypopnea index 9.1/h with average oxygen saturation 96%). Finally, during REM sleep an excessive amount of tonic chin electromyogram activations was evident (Fig. 1), and complex behaviors were also detected during REM sleep through video. During the next-day MSLT, the patient fell asleep in 4 of the 5 sessions with a mean sleep latency of 12.6 min; no sleep-onset REM episodes were recorded.

**Fig. 2:**
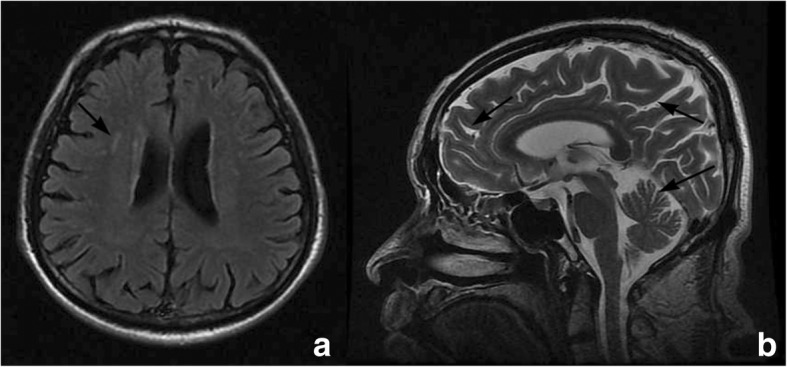
MRI showed a demyelination in right corona radiata (arrow in 2a), with mild cortical and cerebellar atrophy (arrows in 2b)

**Fig. 3:**
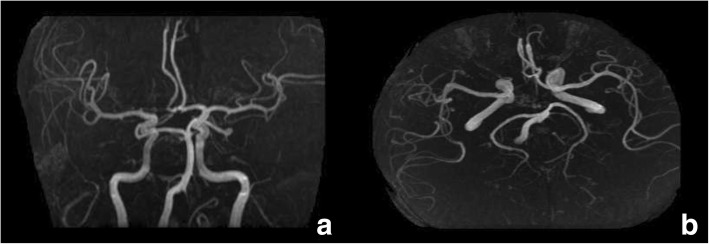
MRA showed a normal cerebral vessel

Thyroid hormones and numerous laboratory blood tests (including iron metabolism) were within the normal limits and a higher titer of ANA (1:80) with positive anti-SSA was detected. Cerebral magnetic resonance imaging (MRI) showed demyelination in right corona radiata, with mild cortical and cerebellar atrophy (Fig. 2) and magnetic resonance angiography (MRA, Fig. 3) showed a normal cerebral vessel. Arterial ultrasound showed no stenosis nor atherosclerosis plaque in the bilateral carotid and subclavian artery. The patient refused a lumber puncture procedure for the assessment of the orexin-1 level in the cerebrospinal fluid.

After a careful consideration of the therapeutic possibilities with the patient and his spouse, an agreement was made concerning therapy and clonazepam was administrated at a start dosage of 1.5 mg at bedtime with pramipexole 0.125 mg/day, which was followed by a beneficial effect on the nocturnal complex motor behaviors and dream-enactment, also had an excellent on discomfort in the lower limbs during sleep and excessive daytime somnolence. Auto set-continuous positive airway pressure (APAP) was also used at the pressure ranging from 5 cm to 20 cm H_2_O to the mild OSA, with the apnea/hypopnea index 0.6/h after APAP treatment.

The clinical history and video-PSG findings satisfied the ICSD-3 (International Classification of Sleep Disorders, 3rd Edition) diagnostic criteria for RBD, mild OSA and severe periodic leg movement disorder (PLMD).

## Discussion and conclusions

In addition to the neurodegeneration associated with RBD in middle or older age patients [4–7], some case reports indicate that inflammatory or autoimmune mechanisms may be associated with RBD, particularly in younger patients [13]. Some cases have structural lesion in the pontine resulted from inflammation, leading to abnormal REM sleep atonia mechanism, which can be commonly found in multiple sclerosis [13]. RBD can also occur in the inflammatory syndrome in central or peripheral nervous system without brainstem involved, including voltage-gated potassium channel antibody associated limbic encephalitis [14], Guillain-Barre syndrome [3, 9, 15] and anti-Ma antibody paraneoplastic encephalitis [16]. Aseptic meningitis with limbic cortexes involved [17] and inflammatory lesion in brainstem [18] have been reported in two separated cases resulting in RBD. Another report suggested that parkinsonism and RBD may be observed in anti-NMDAR encephalitis [19]. Comparing anti-locus coeruleus antibody from RBD patients with normal controls showed no significantly different [20]. The reported autoimmune disorders linked with RBD are listed in Table 1.

**Table 1:** The reported autoimmune disorders linked with RBD and other disorders

Authors	Article	Primary disorder	Other disorders
Iranzo A, Graus F, Clover L, et al.	Rapid eye movement sleep behavior disorder and potassium channel antibody-associated limbic encephalitis.	Potassium channel antibody-associated limbic encephalitis	
Cochen V, Arnulf I, Demeret S, et al.	Vivid dreams, hallucinations, psychosis and REM sleep in Guillain-Barré syndrome.	Guillain-Barré syndrome	
Compta Y, Iranzo A, Santamaria J, et al.	REM sleep behavior disorder and narcoleptic features in anti-Ma2-associated encephalitis.	Anti-Ma2 antibody associated encephalitis	Narcoleptic features
Lin FC, Liu CK, Hsu CY.	Rapid-eye-movement sleep behavior disorder secondary to acute aseptic limbic encephalitis.	Acute aseptic limbic encephalitis	
Limousin N, Dehais C, Gout O, et al.	A brainstem inflammatory lesion causing REM sleep behavior disorder and sleepwalking (parasomnia overlap disorder).	Acute inflammatory rhombencephalitis	Parasomnia (NREM)
Çoban A, İsmail Küçükali C, Bilgiç B, et al.	Evaluation of Incidence and Clinical Features of Antibody-Associated Autoimmune Encephalitis Mimicking Dementia.	Anti-NMDAR encephalitis	Parkinsonism
Adams C, McKeon A, Silber MH, et al.	Narcolepsy, REM sleep behavior disorder, and supranuclear gaze palsy associated with Ma1 and Ma2 antibodies and tonsillar carcinoma.	Anti-Ma1 and Ma2 antibody associated paraneoplastic disorder	Narcolepsy, supranuclear gaze palsy
Cardoso Vale T, Bizari Fernanes do Prado L, Fernnades Do Prado G, et al.	Rapid eye movement sleep behavior disorder in paraneoplastic cerebellar degeneration: improvement with immunotherapy.	Paraneoplastic cerebellar degeneration	
Gomez-Choco M, Iranzo A, Blanco Y, et al.	Prevalence of Restless Legs Syndrome and REM Sleep Behavior Disorder in Multiple Sclerosis.	Multiple sclerosis	Restless legs syndrome
Plazzi G, Montagna P.	Remitting REM sleep behaviour disorder as the initial sign of multiple sclerosis.	Multiple sclerosis	
Tippmann-Peikert M, Boeve BF, Keegan BM.	REM sleep behavior disorder initiated by acute brainstem multiple sclerosis.	Multiple sclerosis	
Sabater L, Gaig C, Gelpi E, et al.	A novel non-rapid-eye movement and rapid-eye-movement parasomnia with sleep breathing disorder associated with antibodies to IgLON5: a case series, characterisation of the antigen, and post-mortem study.	Anti-IgLON5 antibody associated encephalitis	Parasomnia (NREM), sleep breathing disorder

In our patient, the coexistence of two autoimmune diseases, Behcet’s disease and Sjogren’s syndrome, seem to responsible for the abnormal levels of detected auto-antibodies, especially the high titer of ANA and positive anti-SSA. Both of the two auto-antibodies are involved in the autoimmune inflammatory pathology to neuronal and small vessel lesions in CNS. Meta-analysis showed that HLA-DQ and HLA-DR allelic genes (DQA1*0501, DQB1*0201 and DRB1*0301) were found to be risk factors for Sjogren’s syndrome [21]. Especially in Chinese Han population, HLA-DPB1, HLA-DRB1, HLA-DQB1, HLA-DRA and HLA-DQA2 were reported to associate with Sjogren’s syndrome [22]. Schenck CH et al. found RBD appeared to be strongly associated with specific HLA class IIgenes [23]: DQw1 in men with RBD was significantly more than DQw1 rate found in control group. It is suggested that the same HLA allelic genes might overlap with Sjogren’s syndrome and RBD, which indicate a possible shared auto-immune pathophysiological in the pathogenesis of both diseases. Mild cortical and cerebellar atrophy were observed in our patient, with cognitive dysfunction suggested by the reduced MMSE and MoCA scores, especially the cerebellar atrophy could be mediated by auto-antibodies in many cases [24]. The MMSE and MoCA findings would encourage extensive neuropsychological testing to better delineate the cognitive dysfuntion. RBD is common in the elderly population and is significantly related to α-synucleinopathy. Combining the decline in neuro-cognition and mild cortical atrophy, presentation of RBD in this patient could indicate an underlying α-synucleinopathy neurodegenerative disorder in the future. Considering the role of inflammation in the pathogenesis of α-synucleinopathy [25] and the same HLA allelic genes in RBD and Sjogren’s syndrome, it is suggested that RBD may share common physiological process with Sjogren’s syndrome which is related to neuroinflammation.

Also, there were other disorders in this patient. The patient had obvious episodes of leg discomfort during nocturnal sleep with excessive daytime sleepiness, but the mild OSA and negative MSLT findings did not support any other hypersomnia diseases. It is suggested that this patient suffered from PLMD and the somnolence in daytime was due to frequent periodic leg movement during sleep, which were managed after treatment of pramipexole. Also the weakness in the left limbs and deep tendon hyperreflexia in the left lower limb indicated a lesion in the right pyramid tract, which can be verified by a demyelination in right corona radiata in Fig. 2. The demyelination in right corona radiata may be correlated with age or the autoimmune pathology in small vessels.

RBD may have been represented as a possible latent autoimmune process, especially since previously reported cases of RBD have been associated with autoimmune inflammation, therefore it is suggested an immune basis (either primary or secondary) for the RBD. Nevertheless, given the patient’s age (68 years), male gender, his reduced cognitive scores on the MMSE/MoCA, and the well-documented strong association of RBD with α-synucleinopathy neurodegenerative disorders in male patients in his age group, a major open question concerns the possible presence of an underlying synucleinopathy as another predisposing factor (of uncertain degree) for his RBD. Clearly the patient needs careful longitudinal neurological and rheumatological follow-up evaluations to help clarify this question. Finally, postmortem brain histopathological examination can provide definitive answers.

## Abbreviations

ANA: Antinuclear antibody
CNS: Central nervous system
NMDAR: N-methyl-D-aspartic acid receptor
PSG: Polysomnographic
RBD: REM sleep behavior disorder
